# Ampullary adenocarcinoma – differentiation matters

**DOI:** 10.1186/1471-2407-8-251

**Published:** 2008-09-01

**Authors:** Peter Schirmacher, Markus W Büchler

**Affiliations:** 1 Department of Surgery, University Hospital, Im Neuenheimer Feld, 69120 Heidelberg, Germany; 2 Institute of Pathology, University Hospital, Im Neuenheimer Feld, 69120 Heidelberg, Germany

## Abstract

The periampullary region gives rise to two main subtypes of adenocarcinoma that show either pancreatobiliary or intestinal differentiation. New data demonstrates that the histological subtype – more so than the anatomical location – is an important independent prognostic factor. This fuels the discussion about maintaining ampullary cancer as a separate entity.

Junctions between two different types of epithelial lining do not only give rise to unique types of diseases, but are also interesting and relevant areas with regards to tumorigenesis. Typically, carcinomas arising in these areas may show a differentiation resembling either of the colliding epithelia (and sometimes a mixture or an intermediate of both). Especially in larger tumors, this adds to the difficulties in defining the exact anatomical origin of a given tumor.

The ampulla of Vater is one of these epithelial junctions, but its tumors have gained considerably less attention, which is probably due to their relatively low number, their reduced diagnostic accessibility, and the fact that differentiating clinical concepts with regards to neoadjuvant or adjuvant treatment strategies are lacking so far. Surgical treatment of ampullary cancer reaches curative rates of above 50% whereas the treatment of pancreatic cancer still has a very low cure rate of around 10% [1,2]. In particular, lymphatic involvement has been described as limiting the curative possibilities [3] and recently different molecular changes in metastasis associated genes have been shown in ampullary and pancreatic cancers [4]. Thus, from a clinical point of view, surgeons have found that ampullary cancer prognosis also depends on other factors besides resectability, TNM staging, and lymphatic involvement.

It was first described by Kimura and coworkers in 1994 [5] that adenocarcinomas of the ampulla, which comprise 90% of all its malignancies, are constituted of two main histological subtypes, the intestinal and the pancreatobiliary subtypes. This can be attributed to the fact that bile and pancreatic duct epithelia meet the duodenal mucosa. Over the years several other studies have confirmed the findings of the original publication. While the intestinal type is indistinguishable by histological means from its colonic counterpart, the same is true for the pancreatobiliary type when compared to its relatives [6]. Their differences do not reside only in the histological pattern of the tumor cell population. There is sufficient data that show that, despite the common anatomical location, the different subtypes are associated with different premalignant lesions, cell type specific marker and oncogene expression, modes of tumor spread, and extent of and interaction with the extracellular matrix. In summary: both subtypes are likely to have very different molecular pathogeneses.

The paper by Westgaard and coworkers [7] addresses the question of whether in resected periampullary cancer (which includes also very distal bile and pancreatic duct carcinomas and periampullary duodenal cancers) localisation to one of the four compartments or the histological subtyping is more relevant. The study is significant in different ways: Firstly it is prospective, thus carrying less bias, and all consecutive resection specimens have been subjected to a standardized protocol. Secondly, the number of cases is sufficiently large for the purpose of the study and, for comparison, the authors additionally resort to a retrospective collection of almost equal size. Finally the data convincingly support the power of histological analysis. They demonstrate that histological subtyping of (peri)ampullary carcinoma is a significant prognostistic factor (more so than the plain anatomical location), with the intestinal type showing a much better prognosis compared to the pancreatobiliary type. This result is clear cut and important but it comes as no surprise.

It has long been known for adenocarcinomas of other organ sites (especially the stomach) that intestinal type adenocarcinoma carries a more favourable prognosis compared with the other subtypes and, as mentioned before, retrospective data on ampullary carcinomas have shown the same. Furthermore, the ductal type adenocarcinomas of the pancreas or distal bile ducts show an extremely poor prognosis that has been attributed to their disseminating growth pattern, extreme tendency for perineural sheet invasion, and their inaccessibility for systemic therapeutics, potentially due to their extensive desmoplastic stromal reaction.

So, where is the true impact of the current study of Westgaard et al.? It is one more proof that even in the era of molecular marker definition, solid histological pattern analysis is able to develop useful and powerful predictive information. With regards to clinical decision making, preoperative biopsy gains another function, and the future planning of the operation may be further influenced (excision range, resection margins). When it comes to adjuvant and neoadjuvant concepts, the data lend further support to linking ampullary carcinoma to the respective adenocarcinoma types in the adjacent organs and not treating it as a separate entity due to the presumed anatomical localisation. Finally, if differentiation matters more than anatomical location, as proposed by the study, it further erodes the necessity for an independent TNM-classification of ampullary carcinoma and joins other arguments about the difficulty differentiating it from distal bile duct and pancreatic adenocarcinoma, and the fact that many patients even lack a defined ampulla.

Certainly there is a lot more work to do: When it comes to the molecular pathogenesis, the histological subclassification is a solid basis to analyse the presumably different pathogenetic mechanisms especially with regard to the ampullary location. Extended molecular fingerprint analyses may help to answer the questions of whether the pancreatobiliary type of ampullary carcinoma is truly nothing else but a very distal pancreatic/biliary adenocarcinoma and whether the intestinal type should be seen as a plain duodenal cancer. What about the approximately 15 percent of ampullary adenocarcinomas that do not fit in one of the two categories? With these findings in hand, questions of etiology, epidemiology, and preventive measures may be addressed more specifically. Finally based on these results we have to answer the questions of whether TNM-classification has to be adjusted and whether ampullary carcinoma should still be seen as a separate entity in the future.

## Pre-publication history

The pre-publication history for this paper can be accessed here:


